# Supplementary material for: Canola with Stacked Genes Shows Moderate Resistance and Resilience against a Field Population of Plasmodiophora brassicae (Clubroot) Pathotype X
Source: Plants (Basel). 2023 Feb 6;12(4):726. doi: 10.3390/plants12040726 (PMC9960129; doi:10.3390/plants12040726)
Supplement: Supplementary file 1 [file plants-12-00726-s001.zip › plants-2180380-supplementary.pdf]

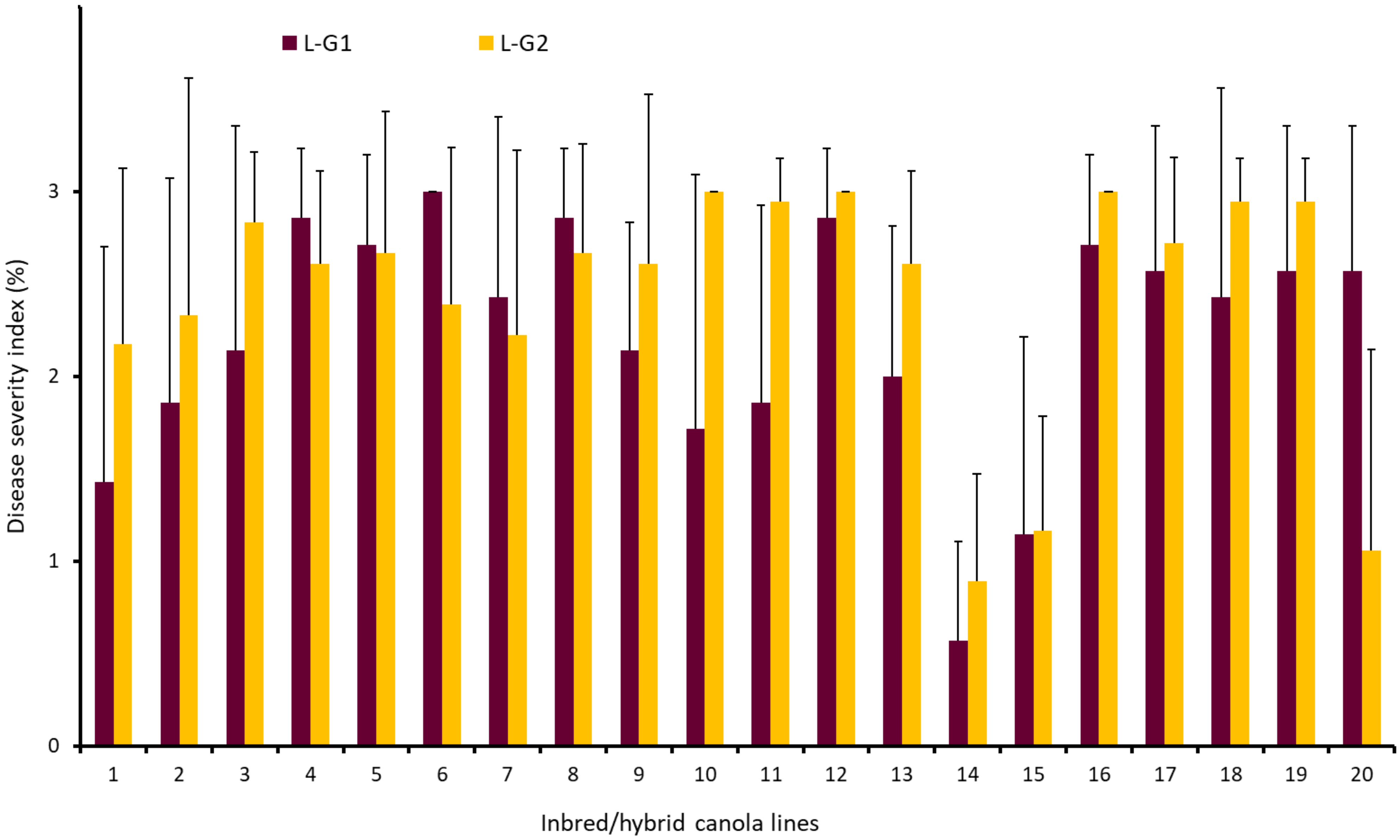

Figure S1. Mean clubroot severity rating (0-3) of 20 inbred/hybrid canola lines carrying different numbers/combinations of CR genes in response to L-G1 and L-G2 of Plasmodiophora brassicae pathotype X. The mean severity ratings on cvs. Westar and 45H29 ranged from 2.7 to 2.9.

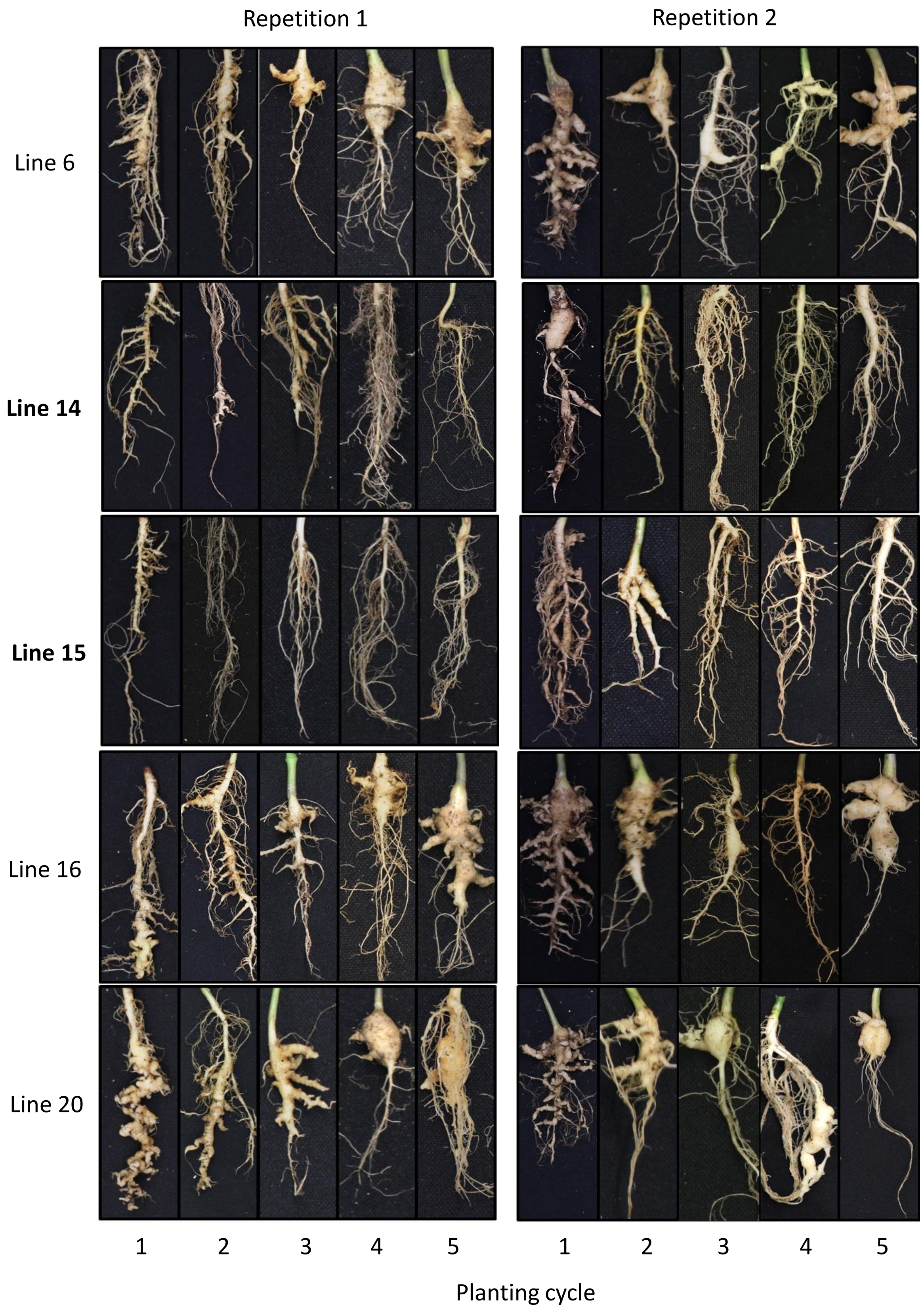

Figure S2. Representative clubroot symptoms for five selected canola lines carrying a single or double CR genes in response to L-G3 of pathotype X of Plasmodiophora brassicae in each of five planting cycles of two study repetitions.
